# Long term movements and activity patterns of an Antarctic marine apex predator: The leopard seal

**DOI:** 10.1371/journal.pone.0197767

**Published:** 2018-06-05

**Authors:** Iain J. Staniland, Norman Ratcliffe, Philip N. Trathan, Jaume Forcada

**Affiliations:** British Antarctic Survey, Natural Environmental Reach Council, Cambridge, Cambridgeshire, United Kingdom; Wildlife Conservation Society Canada, CANADA

## Abstract

Leopard seals are an important Antarctic apex predator that can affect marine ecosystems through local predation. Here we report on the successful use of micro geolocation logging sensor tags to track the movements, and activity, of four leopard seals for trips of between 142–446 days including one individual in two separate years. Whilst the sample size is small the results represent an advance in our limited knowledge of leopard seals. We show the longest periods of tracking of leopard seals’ migratory behaviour between the pack ice, close to the Antarctic continent, and the sub-Antarctic island of South Georgia. It appears that these tracked animals migrate in a directed manner towards Bird Island and, during their residency, use this as a central place for foraging trips as well as exploiting the local penguin and seal populations. Movements to the South Orkney Islands were also recorded, similar to those observed in other predators in the region including the krill fishery. Analysis of habitat associations, taking into account location errors, indicated the tracked seals had an affinity for shallow shelf water and regions of sea ice. Wet and dry sensors revealed that seals hauled out for between 22 and 31% of the time with maximum of 74 hours and a median of between 9 and 11 hours. The longest period a seal remained in the water was between 13 and 25 days. Fitting GAMMs showed that haul out rates changed throughout the year with the highest values occurring during the summer which has implications for visual surveys. Peak haul out occurred around midday for the months between October and April but was more evenly spread across the day between May and September. The seals’ movements between, and behaviour within, areas important to breeding populations of birds and other seals, coupled with the dynamics of the region’s fisheries, shows an understanding of leopard seal ecology is vital in the management of the Southern Ocean resources.

## Introduction

The movement of individuals influences a wide range of important ecological factors including intrinsic aspects, such as population processes and dynamics, and extrinsic elements such as biodiversity, nutrient distribution and the spread of disease[1]. The patchy distribution of resources and separation of habitats, suitable for different activities such as feeding and breeding, are thought to drive large scale animal movements. These long distance migrations can be dangerous and are energetically expensive, however, they allow access to resources that are seasonally or spatially limited but relatively predictable in space and time[1,2].

Whereas many terrestrial migrations have been studied for centuries, our understanding of marine movements was limited. Recent advances in remote telemetry and biologging systems have resulted in a rapid expansion in marine tracking studies allowing the movements of more cryptic animals to be resolved over long time periods. This has allowed us to elucidate the migration patterns of marine predators such as the almost pole to pole movements of Arctic terns [3] and the 22,500 km migration movements undertaken by western gray whales [4].

The role of apex predators in ecosystems is generally multi-faceted, but also difficult to fully determine as they tend to be sparsely distributed with large geographical ranges and complex life histories [5]. In the marine Antarctic, leopard seals (*Hydrurga leptonyx*) are an important apex species with a generalist diet, consisting predominately of krill, fish, seals and penguins. They can affect marine ecosystems both through direct and indirect means, exerting top-down control on fur seal [6,7] and penguin [8,9] breeding, whilst also predating directly on krill. The influence of leopard seals on regional krill stocks is complex as they directly graze krill, but also consume other krill predators [5]. In order to better understand these effects, we need to determine the factors influencing the distribution of leopard seals and their year round movements.

Leopard seals have a circumpolar distribution concentrated in, but not limited to, the Southern Ocean. They are most commonly found in and around the outer fringes of the pack ice or close to the Antarctic Continent [9–11]. Their range extends beyond the Polar Front with regular sightings in South America [12,13], and individuals reported in South Africa [14], Australia [15] and as far north as the Cook Islands [14]. Across this north-south distribution there is evidence of a gradient in age classes with the proportion of younger immature animals increasing in lower latitudes [16]. Although they have been observed year-round on Heard Island [17] and South Georgia [18] their occurrence on sub-Antarctic islands is thought to be mostly driven by a proportion of the population migrating northwards during the austral winter [19–21]. However, the full migration between sub-Antarctic islands and the Antarctic pack ice has never been recorded by tracking individual seals with telemetry.

Leopard seals at Bird Island, South Georgia, have been intensively studied since 1983 revealing a seasonal population consisting of two different types of individuals. The majority (68%) of sightings are of transient juvenile animals that arrive late in the season and remain for less than a week. The remainder are of mature adult individuals that tend to arrive early in the winter and are resident for longer ($\bar{\text{x}}=27$ days, [22]). Locally they predate on krill, fur seals and penguins including a significant proportion (16–20%) of the relatively small gentoo population[5].

In order to assess the role of this highly mobile predator within the Antarctic/Scotia Sea ecosystem we set out to understand its distribution, movement, haul out and residency patterns. Seals are typically censused based on visual counts whilst ashore, or on sea ice, thus quantifying their haul out behaviour is vital for robust population estimates [23]. This behaviour also has important repercussions for animals’ activity budgets and energetics and is driven by a number of factors including rest, thermoregulation, predator avoidance, moulting, pupping, lactation and social interaction. It has been shown to be influenced by environmental factors. Previously leopard seal haul out has been estimated through visual observations, underwater acoustic activity and through biotelemetry [9,24]. Whilst biotelemetry has allowed the full 24 hour cycle to be examined, to date, this has only been possible for parts of the year[25].

Here we report on the successful use of micro global location sensor data loggers (GLS) recording leopard seal movements and activity over extended periods to understand: 1) Where do leopard seals observed at sub-Antarctic islands originate from? 2) What are the environmental conditions that these migrating animals are associating with? 3) Do the timings of arrival and departure correspond to visual based observations? 4) What are the spatial and temporal patterns of haul out behaviour?

## Methods

All animal procedures were approved by the British Antarctic Survey Animal Welfare Board. Micro global location sensor data loggers (GLS, MK 7, 7 x 20 x 19 mm, mass < 4 g) were deployed on leopard seals at Bird Island, South Georgia (54.01°S and 38.05°W) during the austral winter. The GLS were fixed to a Dalton jumbo roto tag and attached to the inter-digital webbing of the hind flipper so that the GLS protruded beyond the edge of the webbing. Loggers were fixed to the Dalton tags using epoxy adhesive and cable ties prior to their attachment to the animals. The leopard seal was approached whilst hauled out and sleeping, and the tag deployed using specialist application pliers. Animals were sexed and the standard length (nose to tip of tail) estimated by measuring adjacent to the sleeping animal.

Loggers measured light levels every 60 seconds recording the maximum light level in a 10 minute period [26]. Salt-water immersion was assessed every 3 s and the number of positive saltwater tests in each 10 min interval recorded: an immersion value of 0 indicates that the logger was completely dry and 200 that it was completely wet.

Tags were recovered at Bird Island the following winter when an animal was sighted hauled out and asleep in an accessible location. The cable ties holding the logger to the tag were cut and the logger removed. GLS devices were calibrated for one month being fixed in a known position with a full solar aspect before deployment and after recovery. After calibration the tags were downloaded and the data decoded using Bastrack software (British Antarctic Survey, Cambridge UK).

### Data analysis

Times of sunrise and sunset were determined in the BAStag R package [27,28] in an interactive process using calculations based on a user defined solar zenith at twilight and a threshold light level. Values for zenith angle and the light threshold were determined for each deployment using calibration data collected when the tag was fixed at a known location with a full view of the sky on Bird Island prior to and post deployment.

The probability of observing a recorded light level at a given location and time was calculated using a two pass recursive algorithm combining forward/backward sweeps (SGAT R package[29]). Days when the animal was observed on Bird Island were fixed as known locations. The model also included a movement constraint chosen so that the mean speeds between successive locations were independently log-Normally distributed. We used a mean swim speed of 1 km h^-1^ with a variance of 0.9kmh^-1^ taken from animals tracked with PTT devices (Rodgers pers. comm.).

An initial path was calculated that balanced the likelihood of a position and the probability of the transition. The posterior was approximated using Markov Chain Monte Carlo (MCMC) method. After initialization using a coarse grid of locations the MCMC proceeded by generating a direct summarization of the posterior (estimated locations). These estimates were binned and quantiles were calculated to give the most probable sampled locations and time spent maps generated from intermediate location estimates. This approach can generate a range of estimated locations, for each seal at each point in time, based on the original calculated position that also allow for realistic speed and distance metrics. These estimated locations reflect the uncertainty surrounding the animal’s exact position and can be passed into subsequent analyses thus reducing the effects of the inherent lower accuracy of using geolocation.

Between the 20^th^ November 2012 and the 22^nd^ January 2013 B4943 experienced 24 hours daylight so that its position could not be determined. For the analyses of habitat association and haul out probability, its last known position was used.

### Habitat association

To determine the animals’ associations with their environment we compared estimated locations to those of a null model generated from a random walk based on the mean trajectory and step distances, taken from the MCMC analysis, using the adehabitatLT package in R [30]. Given the relatively low accuracy of our location data we accounted for this uncertainty by using 100 MCMC chains taking the mean of each environmental parameter at each time interval and comparing with values taken from 10 runs of the null model. Bathymetry, sea surface temperature and sea ice concentration at the estimated locations were compared to the null model using an adaptation of the approach outlined in Wakefield et al. [31]. We modelled the probability of an animal occupying a particular space as a function of the environmental covariates using GAMMs with a binomial response and a logit link function [32]. Penalized smooths were fitted to covariates using cubic regression splines with shrinkage so that simple linear relationships could be selected were appropriate. Models were compared using forward selection based on maximizing the log-likelhood. Sea surface temperature (SST) values were taken from the NOAA optimum interpolation analysis version 2 [33]. Weekly mean values were used where possible with the monthly mean as alternative where the relevant weekly value was missing. Sea ice concentrations were calculated from the University of Bremen archives using the Artist sea ice algorithm based on the Advanced Microwave Scanning Radiometer [34]. Bathymetry values were taken from GEBCO_2014 grid (30 arc-second interval) and all values where the animals were hauled out on land were discarded.

### Haul out

To determine haul out periods we used a modification of the approach outlined in Staniland et al. [35]. Immersion data from all loggers were processed in R, using methods from the diveMove package [36] to objectively identify the start and end times of each dry period. Immersion readings were first converted to zero or negative values by subtracting 200, such that potential haul out periods now appeared as ‘dives’ to the analysis package. Haul out duration was measured between when the sensor first became completely dry (-200) until it again became wet (>-200). Only dry periods during which the logger was completely dry for at least 1 hour were analysed. Each hour of the animal’s deployment period was assigned as haul out or at sea depending if there was any overlap with an assigned dry period. We analysed the relationship between hourly haul out behaviour and covariates using a GAMM (gamm4 package R [32]). As these data were binomial we used a complimentary log-log link function chosen as it performs better than logit where one response is relatively rare. We used hour of the day to measure diel effects and tested month, week or Julian day to model seasonal effects. All were fitted with a cyclic smooth. Distance from land or ice was measured between the animal’s estimated location and the closest part of the nearest land polygon using the haversine formula based on worldmap data (R package maptools) or the monthly median ice extent [37]. We tested fitting separate smooths for each seal or month for these terms. The effect of latitude was tested using seal, month or week as different grouping terms.

To account for auto correlation within the data we tested terms as to whether the seal was hauled out in the preceding hours with a separate one for each hour. Individual seal was used as a random effect because of the repeated measures nature of the data with trip nested within tag, to account for the two trips by seal Y5282. At each stage the selection of terms to be dropped, or alternative grouping terms to be tested, were guided by the approximate significance of the terms and the model outputs were compared using AIC and examining residual plots.

## Results

Thirty-one deployments were made between 2003 and 2010 with 5 tags recovered (~16%, up to 2016). Of the rest, 15 seals were never seen again, eight were observed but with lost flipper tags and three observed with tags but inaccessible. Whilst a sample size of five trips from four individuals is small the longest record lasted 466 days (Table 1) with shortest lasting 142 due to tag failure. One seal (Y5282) was tracked in two separate periods just under eight years apart.

**Table 1:** The biometrics, timings and summary of data from leopard seals deployed with micro global logging sensor tags.

Tag	Sex	Length nose to tail /cm	First observation	Last observation	Years observed	Date deployed	Date recovered	duration (days)	max. distance reached (km)	Date of departure South Georgia	Date of return South Georgia	haul out duration				Inter haul out period (mins)		
												total time (days)	proportion of time	max (hours)	median (hours)	median interval	min interval	max interval
Y5282 (B4942)	M	285	01/09/1993	14/06/2013	15	18/05/2003	26/08/2004	466	1950	09/06/2003	02/05/2004	102	0.22	34	10	14	20	326
Y5282 (B4942)						24/07/2012	13/06/2013	324	1500	28/08/2012	16/05/2013	76	0.23	40	9	14	30	600
B4943	M	278	01/09/2011	18/04/2016	6	02/08/2012	09/05/2013	280	1850	13/10/2012	21/04/2013	76	0.27	47	10	14	20	363
G5719	F	273	07/07/2011	28/08/2012	2	07/07/2011	09/06/2012^a^	142	800			31	0.22	30	11	13	20	353
W7604	M	236	16/07/2006	28/08/2012	4	07/09/2007	24/05/2008	260	1850	02/10/2007	23/05/2008	81	0.31	74	11	12	30	604
							mean	294.4				mean	0.25					

^a^ Tag failed on 26/11/2011

**Fig 1:**
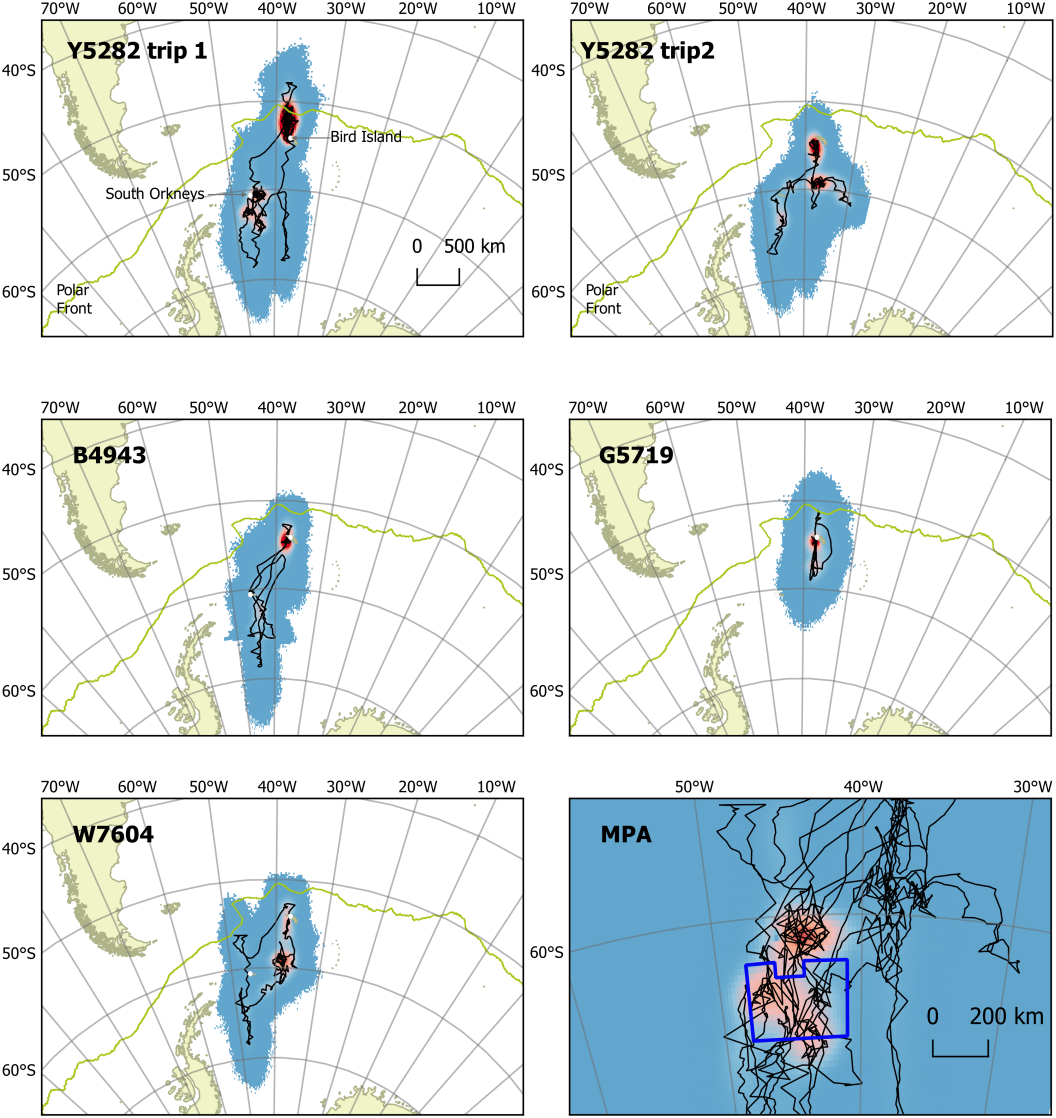
Density of time spent by individual leopard seals estimated by geolocation. Each plot shows the gridded output of 1000 simulations of the intermediate location estimates weighted by time from low (blue) to high (red). Black lines show the mean estimated track. The pale green line indicates the approximate position of the polar front. MPA panel shows the outline of the South Orkney Islands’ marine protected area (thick dark blue line) with the combined time spent grid and mean estimated tracks from all seals.

Previous analyses of GLS devices deployed in tandem with PTT devices on Antarctic fur seals show that both the precision and accuracy of GLS locations are relatively low, with an average error of approximately 183 km [35] although this estimate is based on only using a simple speed filter to determine the animals’ locations. Whilst our Bayesian approach allowed us to account for uncertainty in our analyses these location errors should be borne in mind when considering fine scale movements. All seals appeared to undertake a number of shorter range trips, that varied in distance around a central place on Bird Island: including short trips (~100 km) in an around the shelf break region, or longer trips (~300–500 km) northwards to the Polar Frontal Zone or southwards (Fig 1). In addition, all seals that were tracked over the austral summer undertook long southward migrations to the pack ice including areas in and around the South Orkney islands (Fig 1). The farthest distance reached from the deployment location on Bird Island was 1,950 km (Table 1). All of the seals stayed in and around Bird Island for at least 3 weeks with the longest resident for 72 days before it departed.

### Environmental association

The most parsimonious model showed that leopard seals had an association with areas of shallower water than the simulated positions (Table 2). The next best model based on the maximum log-likelihood indicated that the tracked leopard seals were associated more strongly with areas of sea-ice than the simulated tracks (Table 2) and these two covariates combined in the third best model. Whilst the model using SST showed leopard seals associating with colder temperatures, SST was strongly correlated with both bathymetry and sea ice concentration and the models including this term had lower log-likelihoods (Table 2). None of the models using smooths significantly improved upon the model fit.

**Table 2:** Generalised Additive Mixed effects models to test environmental associations of tracked leopard seals against simulated locations derived from correlated random walks. Log-likelihoods of candidate models are shown and the model estimates of the fixed effects are shown for the three best models.

Environmental covariates	Log-likelihood	d.f.			
Depth	-37641	3			
Ice Concentration	-38559	3			
Sea Surface Temperature	-43809	3			
s(Depth)	-79647	4			
s(Ice Concentration)	-5018268	4			
s(Sea Surface Temperature)	-47452	4			
Depth + Ice Concentration	-38525	4			
Depth + Sea Surface Temperature	-43723	4			
Ice Concentration + Sea Surface Temperature	-43035	4			
Three best performing model outputs					
	Value	Std Error	DF	t-value	P-value
Intercept	1.8525575	0.06117873	15794	30.28107	<0.0001
Depth	0.0001004	0.00001796	15794	5.592335	<0.0001
Intercept	1.8182649	0.04996534	15794	36.39053	<0.0001
Ice Concentration	-0.0120945	0.0003393	15794	-35.6459	<0.0001
Intercept	1.472342	0.6623101	15793	22.2304	<0.0001
Depth	-0.0001054	0.00001423	15793	-7.40215	<0.0001
Ice concentration	-0.0121492	0.00034026	15793	-35.7056	<0.0001

### Haul out

**Fig 2:**
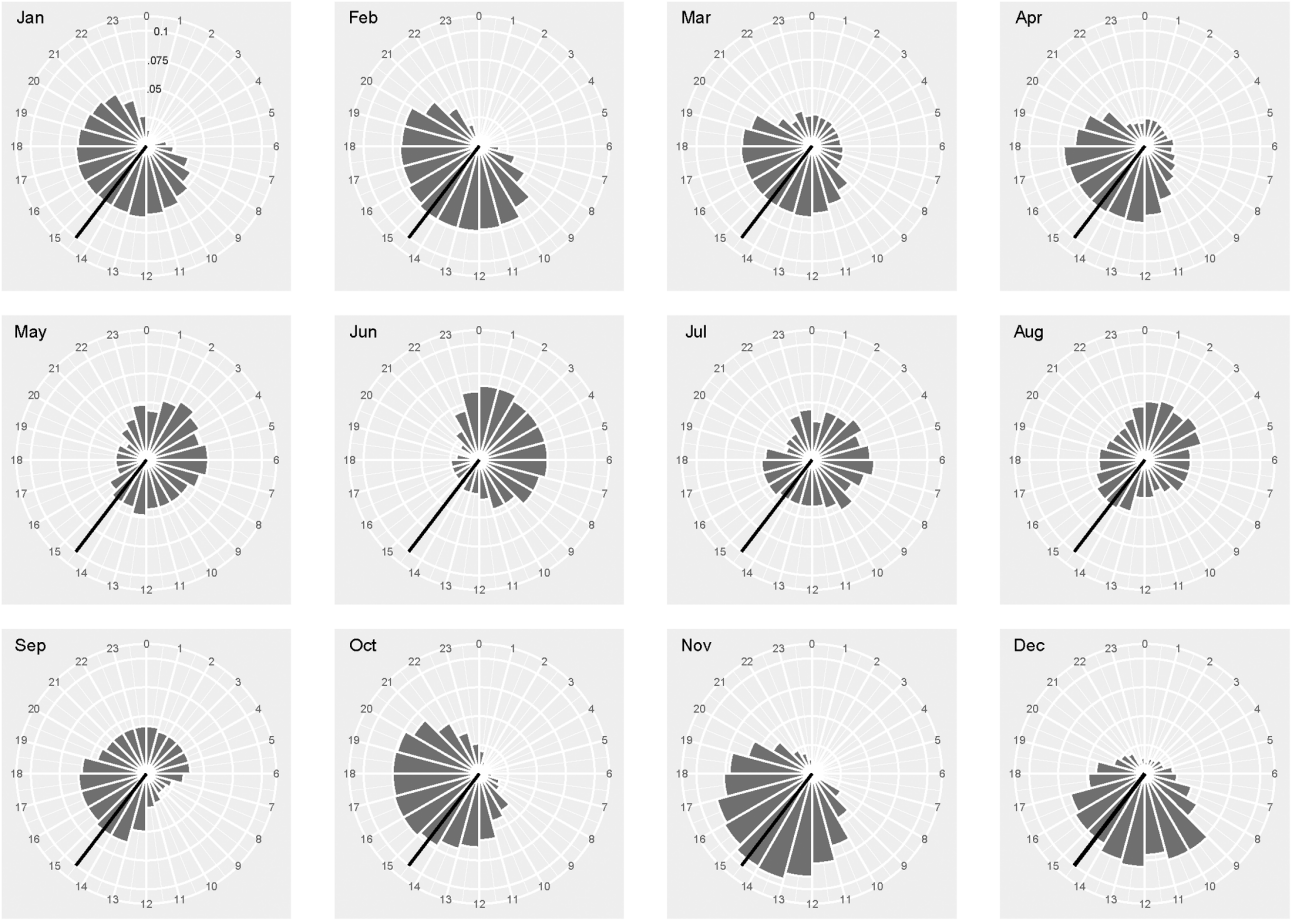
Proportion of time spent hauled out for each hour of the day in each month. The time of local noon at the deployment site (Bird Island, 54.01°S and 38.05°W) is indicated by the black line.

**Fig 3:**
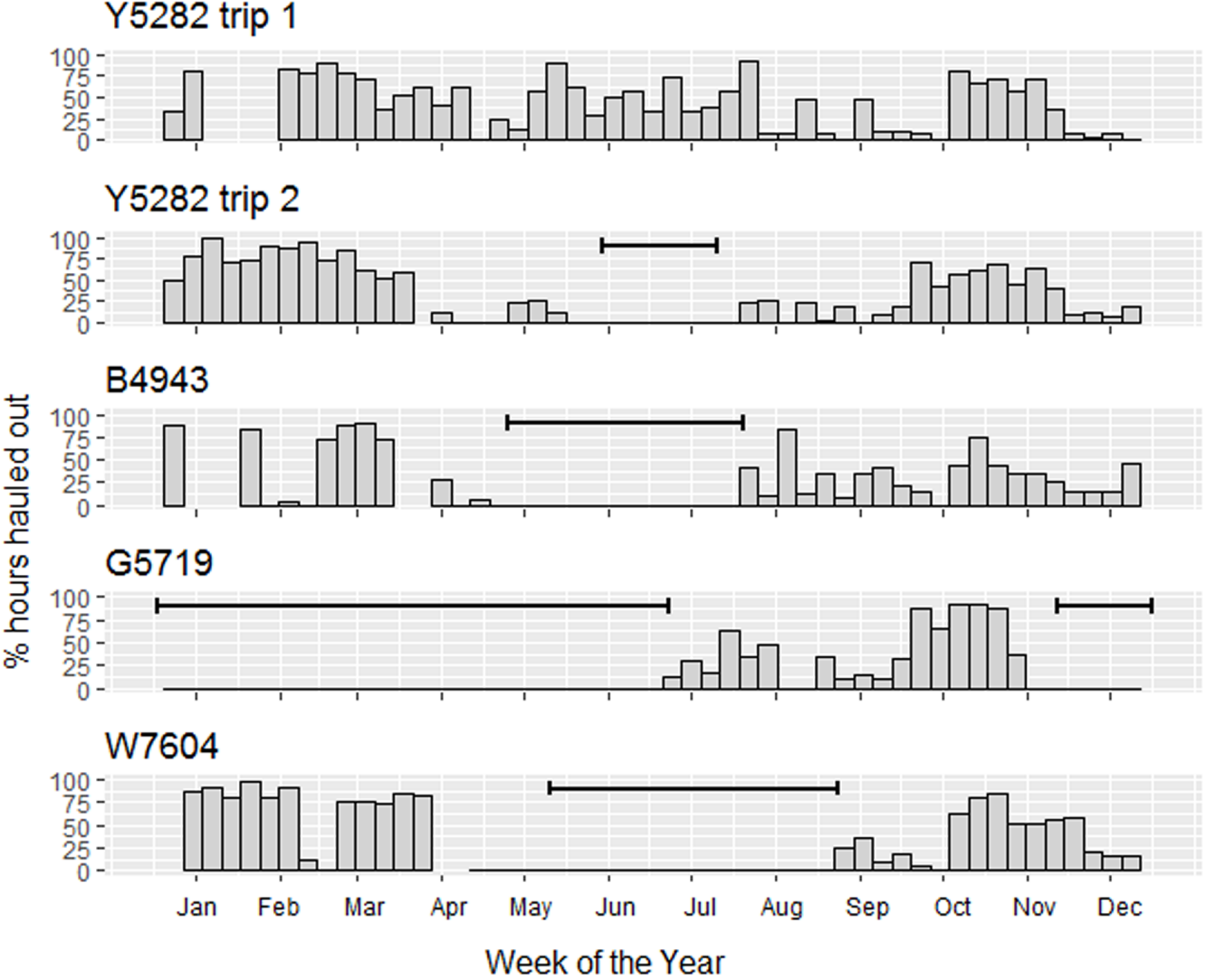
Proportion of time spent hauled out in each week for each individual leopard seal (and trip). Solid black lines indicate periods that were not recorded.

**Fig 4:**
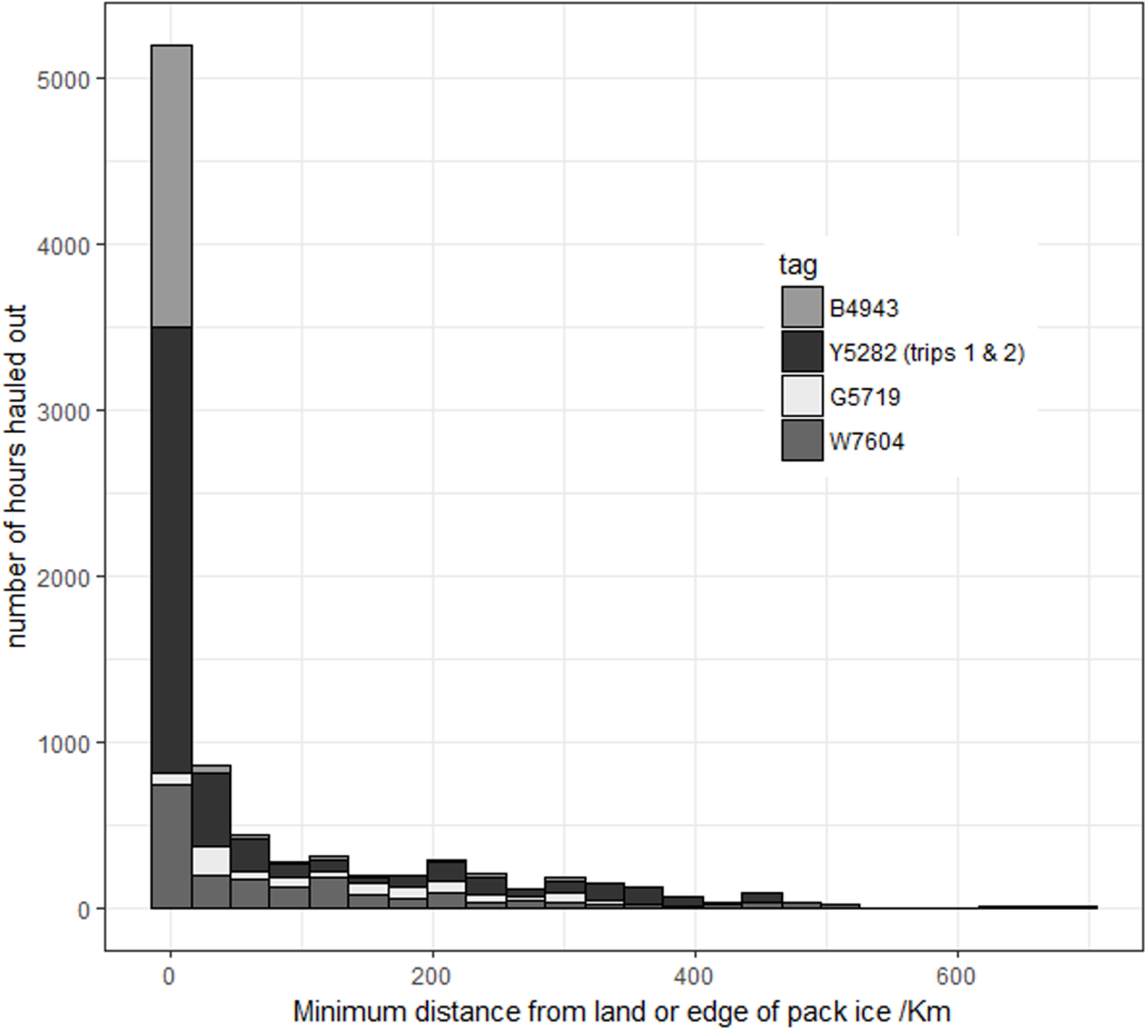
Hours hauled out related to the minimum distance to land or the monthly mean edge of the pack ice.

Seals hauled out for between 22 and 31% of their time (Table 1), the maximum haul out was 74 hours with a median of between 9 and 11 hours. The longest period the seals remained in the water was between 13 and 25 days. The most parsimonious model describing the hourly haul out behaviour of the seals included terms for hour of the day, week, latitude, and distance from land or edge of the pack ice. There was a strong positive relationship with being hauled out in the previous hour but a negative relationship with the preceding 2 and 3 hours. The models including separate smooths of hour of the day for each week performed better than those fitting separate smooths for each individual or month (Tables 3 & 4). Peak haul out occurred around midday for the months between October and April but the probability was more evenly spread across the day between May and September (Fig 2) The probability of an animal hauling out changed throughout the year with the highest levels in summer and the lowest in November except for G5719 who’s haul out peaked in October (although this seal only had a limited recording period, Fig 3). Week provided a better fit than Julian day or month and although individual seals showed significant differences this was driven by W7604 and G5719. Distance from land/ice edge unsurprisingly showed an exponential decay, with differences between the seals, but also suggested that some animals were apparently hauled out in open water (Fig 4). Latitude fitted best when grouped by week reflecting the dynamic nature of the pack ice and the timing of the seals’ migrations.

**Table 3:** Generalized Additive Mixed effects models with the probability of an individual seal being hauled out in each hour of a trip modelled as a binomial response. Individual seal was a modelled as random effect with trip nested within it. AIC values are reported for the best model fit and the effect of adding (+) or removing (-) explanatory variable including different ways of grouping smoothers. Correlation was modelled by including terms of whether the seal was hauled out in the preceding hours (lag1-5). Time of year was compared using Julian day, week of year, month.

Model terms	AIC	BIC	deviance	df.resid
*Best model*				
Hour (by month) + Latitude (by week) + Distance to land/ice (by tag) + week (by tag) + lag1 + lag2 + lag3				
	10525.1	10845.9	10449.1	34222
*Comparison with alternative models*				
+ lag4	10523.1	10853.1	10445.9	34221
-lag3	10533.7	10846.1	10459.7	34223
-distance to land/ice(by tag)	10557.8	10817.7	10497.8	34230
-'(by tag)' from 'distance to land/ice'	10546.2	10821.3	10482.2	34228
-'(by tag)' from 'week'	10539.8	10837.7	10469.8	34225
'month(by tag)' replacing 'week(by tag)'	10610.0	10930.8	10534.0	34222
'Julian day(by tag)' replacing 'week(by tag)'	10553.7	10874.5	10477.7	34222
-'(by tag)' from 'Latitude'	10550.6	10820.8	10486.6	34228
Latitude (by tag)	10873.3	11118.0	10817.3	34232
Latitude(by month)	10486.6	10929.2	10378.6	34206
hour(by Tag)	10794.6	11084.9	10726.6	34226

**Table 4:** GAMM outputs from the best model describing the probability of an individual seal being hauled out in each hour of a trip. Individual seal was modelled as a random effect with trip nested within it.

Parametric coefficients:					
	Estimate	Std. Error	z value	Pr(>|z|)	
(Intercept)	-3.2205	0.143	-22.527	< 2.00E-16	***
lag 1	5.0398	0.1567	32.156	< 2.00E-16	***
lag 2	-0.184	0.1838	-1.001	0.316781	
lag 3	-0.4871	0.136	-3.581	0.000342	***
Approximate significance of smooth terms:					
	edf	Ref.df	Chi.sq	p-value	
hour:Jan	5.68E+00	9	103.189	< 2.00E-16	***
hour:Feb	5.47E+00	9	178.766	< 2.00E-16	***
hour:Mar	4.91E+00	9	50.514	3.78E-11	***
hour:Apr	3.63E+00	9	31.196	1.66E-07	***
hour:May	2.65E-08	9	0	0.32637	
hour:Jun	2.52E-01	9	0.223	0.415131	
hour:Jul	1.61E-02	9	0.007	0.6912	
hour:Aug	3.31E-04	9	0	0.559409	
hour:Sep	2.99E+00	9	11.036	0.005732	**
hour:Oct	4.53E+00	9	96.668	< 2.00E-16	***
hour:Nov	4.57E+00	9	150.562	< 2.00E-16	***
hour:Dec	2.87E+00	9	21.7	1.06E-05	***
Latitude:week	2.00E+00	2.001	33.546	5.21E-08	***
Distance:B4943	2.22E+00	2.223	17.8	0.000125	***
Distance:Y5282	4.16E+00	4.162	34.703	7.03E-07	***
Distance:G5719	1.00E+00	1	0.536	0.464292	
Distance:W7604	1.68E+00	1.677	2.377	0.346307	
Week:B4943	7.02E+00	9	42.824	5.43E-08	***
Week:Y5282	7.38E+00	9	40.977	1.29E-07	***
Week:G5719	4.18E+00	7	53.303	7.66E-12	***
Week:W7604	8.74E-01	9	1.897	0.087296	.

The level of statistical significance is indicated by the codes:

‘***’ < 0.001;

‘**’ < 0.01;

‘.’< 0.1;

‘ ‘ < 1

## Discussion

Our data report the first long-term deployments of tracking devices on leopard seals, that include both winter and summer haul out behaviour. Although the sample size of individuals was small, the length of the deployments means that animals had sufficient time to explore their environment which expands our sampling across the temporal if not individual dimension. However, our sample is biased in that it only represents animals that returned to the tagging site, which is a tactic of a small number of individuals and may be unrepresentative of the wider population across the Weddell Sea and WAP. Leopard seals have proven to be difficult to study, a total of 13 animals have previously been successfully tracked [25,38]. Therefore, although our sample size is small, the results represent an advance in our limited knowledge providing novel insights into the seasonal patterns of movement of one of the true ‘apex’ predators in the Antarctic. Allowing us to consider issues of their movement ecology, including home range and migration. These data show the longest periods of tracking of leopard seals migratory behaviour between the pack ice, close to the Antarctic continent, and the sub-Antarctic island of South Georgia.

The key to the longevity of our deployments was the use of geolocation (GLS) tags that have low power consumption and could be attached to flipper tags, avoiding the issues associated with device loss during the animals’ annual moult and battery drain in devices relying on satellite positioning. Overall recovery rates were relatively low (16% compared to 36% in Antarctic fur seals, [35]) but improved when only individuals that had been seen in previous years (i.e. seasonal resident animals[22]) were considered (31%). We observed no necrosis of tissue, or damage to flippers associated with the long-term deployments. Whilst we could not measure stress in the seals, the fact that the observed animals’ haul out behaviour and residency durations did not change post deployment and recovery suggests that the tags had no detectible negative impacts. Re-sighting rates of deployed animals were comparable to those of the general population, taking into account the two different groups: transient and seasonal residents.

### Use of geolocation

Leopard seals typically dive during crepuscular periods [25,39] and we might expect this to interfere with the determination of sunrise and sunset due to light attenuation at depth. However, leopard seals are short duration (< 5 mins) shallow divers (<50 m) so there is a high chance that the animal will be at the surface, and the correct ambient light level sampled, at least once during each 10-min sampling period of the loggers[25,40]. Indeed issues with shading of the light sensor were most commonly associated with periods when the seals were hauled out and shading the light sensor with their body, similar to issues for deployments on birds’ legs when they are sitting on their nests [41]. Despite the limitations of lower accuracy in estimated locations and the need to recapture tagged individuals, the robustness, small size and lower relative expense coupled with new Bayesian based approaches to geolocation allowed us to resolve the year round movements, habitat associations and haul out behaviour of a cryptic marine predator in an extreme environment.

The complete migration of leopard seals between the Antarctic pack ice and sub-Antarctic islands had previously not been recorded, with a particular paucity of data from winter months. We tracked seals for an average of 294 days with the longest a period of 466 days. Taking advantage of the seasonal site fidelity of some animals we were able to track one individual in two separate years; a total (but not consecutive) duration of 790 days. Two previous studies that used satellite derived locations had issues with high tag failure rates and were limited in the periods they could deploy instruments because of the animal’s moult cycle. The first study to track individual leopard seals had a maximum deployment of 282 days (mean 84 days) and found little in the way of dispersal with all animals staying within the Prydz Bay area and 33 to 320 km of the tagging site [38]. A second study of two seals tagged just off Queen Maud Land successfully tracked one seal for 220 days (80 days for the other seal) that reached 55°S staying within the edge of the expanding pack ice [25].

Using the Bayesian approach to geolocation was successful and through simulations the uncertainty surrounding location estimates could be passed easily into other analyses. This approach also overcomes issues related to the equinox periods such that, whilst resolution of the latitude component is compromised, the model output can reflect the increased errors whilst the movement component provides an additional restriction to possible locations. Even with improved techniques geolocation will always provide a lower accuracy than satellite derived location estimates. Another limitation of geolocation is that it is not possible during periods of 24 hour daylight. This occurred with one seal B4943 for 63 days; however, we do at least know the maximum possible northerly boundary of the animal’s location during this period.

### Movements

From the recorded movements the arrival and residency times of the individuals fit well with the visual observations of these seals on the island. It appears that these animals migrate in a directed manner towards Bird Island and during their residency use this as a central place for foraging trips as well as exploiting the local penguin and seal populations. This behaviour means that land-based observations provide a good estimate of the residency of the animals in the region although our data suggest that the seals are exploiting resources over a larger area than may have previously been considered. After deployment the seals remained at South Georgia for between 22 and 72 days, compared to a mean residency of 27 days calculated from resightings [22]. This suggests that the deployments did not cause animals to abandon the site and there were no detectible effects on their time spent around South Georgia.

The seals associated with both shallower water and sea ice more than would be expected by chance. This fits with the known distribution of the animals and their observed behaviours. By definition these resident seals were spending long periods of time in and around South Georgia and thus are spending significant time in its shallow shelf waters. They also spent time in the waters around the South Orkney Islands further emphasising this association (Fig 1). Given that the prey of these animals, krill, penguins and seals, are typically concentrated in areas around sub-Antarctic islands it is no surprise to find a preference for these areas amongst the seals tracked. Seals observed at Bird Island, South Georgia, preferentially haul out on ice or snow and, when available, will preferentially choose floating ice (British Antarctic Survey unpublished). Given that the bulk of the population resides in the pack ice surrounding the Antarctic continent, and their adaptations to cold conditions, their pagophilic behaviour is expected. The previous tracking of these animals showed an even greater affiliation with the Antarctic pack ice with animals remaining in or around the immediate vicinity. Indeed the abundance of leopard seals at Bird Island is observed to increase in years of lower sea surface temperatures and more extensive pack ice [21]. It is perhaps more surprising that these seals are found so far north with individuals apparently foraging in the Polar Frontal Zone (Fig 1).

The lengths of the animals tracked indicates they were adults rather than juvenile animals. Juveniles are usually associated with a more northerly distribution than adults [16], and the majority of these younger seals that are seen at Bird Island are not seen again and are considered transient [22]. Given the general lack of resightings it would appear that these juvenile animals observed at Bird Island either die or settle on alternative strategies (migration to other areas or residency in the pack ice). Therefore, to track these animals the use of archival loggers is not appropriate as they cannot be recovered. Understanding the movements of this important part of the population will require satellite tagging as these relay positions via the ARGOS system such that acquisition of data is not dependent upon resightings or recapture.

The seals tracked in this study only represent a small proportion of the overall leopard seal population although these seals still potentially have a large local impact. The observed migrations must be beneficial to the individuals concerned presumably allowing them to take advantage of the rich marine resources surrounding sub-Antarctic islands and potentially reducing intra-specific competition with non-migratory individuals that remain in the Antarctic pack ice. Whilst the benefits must outweigh the travel costs it should be noted that, with the northwards movement of the ice, the scale of these migrations is reduced as the distance between South Georgia and the ice edge is typically at a minimum at the time when these animals migrate. Indeed in years of extensive sea ice when travel costs are at their lowest the occurrence of leopard seals at South Georgia is at its highest [21].

### Regional context

The linkages between South Georgia and the South Orkney Islands that were observed in the migration of the leopard seals tracked in this study mirror those of other predators. Male Antarctic fur seals have been tracked from the breeding beaches on Bird Island to the South Orkneys and the seasonal build-up of animals there is thought to be linked to the southwards migration of males from South Georgia after the mating season [35,42,43]. Although it should be noted that leopard seals head in the opposite direction, breeding in Antarctica and spending the post breeding period at South Georgia. This area is also important for procellariform species nesting on South Georgia, in particular incubating and brooding black-browed albatrosses and postbrood grey-headed albatrosses, light-mantled sooty albatrosses, northern giant petrels and southern giant petrels [44]. These links have numerous implications such as the spread of disease and importantly the functioning of the Scotia Sea ecosystem. The South Orkneys are home to large populations of penguin species of which Adélie (*Pygoscelis adeliae*) and chinstrap (*Pygoscelis antarctica*) populations have declined over recent decades [45]. Leopard seal predation has been proposed as a top down control on some species [6,7] and whilst their numbers, compared to other ice seals, are low they can have significant ecosystem effects at the local level [5].

There is also another major consumer of krill that moves between the South Orkney Islands and South Georgia; the commercial krill fishing industry [46,47]. The krill fishery generally operates around the South Orkney Islands during the austral summer moving to South Georgia when the pack ice limits access to more southerly fishing grounds [48]. In addition, the krill fishery at South Georgia does not operate in the summer thus avoiding conflict with the animals breeding on the island. These timings mean that the movements of the fishing fleet essentially mirror those of the leopard seals tracked in this study. Whilst leopard seals consume krill and are thus in direct competition for resources; their diet, particularly of the larger animals, is made up of other krill predators and thus the relationship is more complex. Both South Georgia and the South Orkney Islands lie within the area managed by the Commission for the Conservation of Antarctic Marine Living Resources, where fisheries are regulated using an ecosystem-based management framework. Interestingly the seals in this study are one of the few tracked predators to use the South Orkneys MPA (Fig 1) [44].

### Haul out

The reduced conductivity of air compared to water is a major factor for thermoregulation and energy expenditure in homoeothermic marine animals so that even when the air temperature is below that of the sea (which it often is in the Southern Ocean) hauling out can help reduce heat loss. It also has advantages for predator avoidance, moult, resting, parasite reduction and is vital for pupping in pinnipeds [2]. The seals in this study hauled out for an average of 25% of their time indicating the importance of this behaviour. These values were low compared to the mean 58.2% recorded for leopard seals hunting around Livingston Island on the Antarctic Peninsula in January and February [40]. However, the GAMM model showed that haul out rates changed throughout the year with the highest values during the summer period (Fig 3) which may account for this discrepancy and highlights the importance of long term deployments. The rate of haul out is difficult to assess during the winter months as only one seal was deployed with a tag during this period. Tags were recovered as soon as possible after animals returned, to ensure recovery of their data, meaning mid-winter periods were lacking. However, the pattern appears to hold in that the number of hours of haul out is less during the rest of the year. Interestingly W7604 (weeks 8 & 9) and B4943 (weeks 1 & 6 to 8) both had periods when there was an absence, or near absence, of haul out. This behaviour may be related to recovery of body condition after the moult but was not seen in the two trips of seal Y5282. A low haul out probability was also seen in February for two seals tracked off Queen Maud Land showing this behaviour may be common amongst leopard seals [25]. If this is the case the behaviour has implications for leopard seal population estimates as this is a time when census are typically carried out. Seals can only be seen and counted when hauled out, so census made at this time of year will provide greater underestimates than those at other times unless accurate correction factors can be derived from haul out data such as that presented here.

Previous biotelemetry data have shown that haul out probability was highest at midday and that this changed from 40% up to 80% between February and April [25]. Our data showed a similar pattern of peak haul out around midday for the months between October and April but the probability was more evenly spread across the day between May and September (Fig 2). This most likely reflects the large intra-annual changes in day length experienced by the seals at high latitudes, with full, or near full, 24hr daylight in midsummer. The change of the haul out pattern in winter coincides with their migration north and could be linked to a change in prey or more likely a change in the behaviour of their prey i.e. the non-breeding period for seals and penguins and differential vertical migration of zooplankton/fish.

As expected there was strong autocorrelation in the data with the probability of being hauled out dependant on the state in the preceding 3 hours. The durations showed that seals were often hauled out for long periods (median 9–11 hours) with some lasting over 24 hours. These very long periods were associated with winter at South Georgia and are probably related to the digestion of a large meal or at least resting rather than moulting or breeding. It is also no surprise that distance to land/ice and latitude were also significant terms in the probability of a seals being out of the water. Changes in the effect of latitude throughout the year is most likely related to the dynamic nature of the sea ice and the movements of the seals themselves. Whilst seals mostly hauled out when close to land or the pack ice there were occasions when they appeared to do so in open water. This most likely comes from the seals utilizing floating ice outside of the pack ice zone, however we cannot discount the issues related to errors in the estimated locations of seals and, errors and lags in using mean ice edge positions.

### Conclusions

A comprehensive understanding of the timings of leopard seal movements and the factors influencing their haul out behaviour is important both to enable robust population size estimates and to assess their role in the Southern Ocean ecosystem. Here we have demonstrated the successful year round tracking of individual leopard seals recording for the first time the migration of animals from the Antarctic pack ice to a sub-Antarctic island and their haul out activity. These movements between areas of important bird and seal populations, coupled with the dynamics of the region’s fisheries, means an understanding of leopard seal ecology is vital to the management of the Southern Ocean resources. The behaviour of individuals differed, including the behaviour of one individual across years. This highlights the complex ecological niche occupied by these apex predators and the need to better understand the range of behaviours at an individual and population level.
